# A long non-coding RNA *PelncRNA1* is involved in *Phyllostachys edulis* response to UV-B stress

**DOI:** 10.7717/peerj.15243

**Published:** 2023-05-09

**Authors:** Lu Yu, Yiqian Ding, Mingbing Zhou

**Affiliations:** The State Key Laboratory of Subtropical Silviculture, Bamboo Industry Institute, Zhejiang A&F University, HangZhou, ZheJiang, China

**Keywords:** Phyllostachys edulis, Long non-coding RNA, UV-B stress, Gene expression

## Abstract

*Phyllostachys edulis* (moso bamboo) is China’s most widespread bamboo species, with significant economic and ecological values. Long non-coding RNA (lncRNA) is a type of regulatory RNA that is longer than 200 nucleotides and incapable of encoding proteins, and is frequently involved in regulating biotic and abiotic stress and plant development. However, the biological functions of lncRNA in moso bamboo are unknown. In this study, a lncRNA (named *PelncRNA1*) differentially expressed following UV-B treatment was discovered in the whole transcriptome sequencing database of moso bamboo. The target genes were filtered and defined by correlation analysis of *PelncRNA1* and gene expression pattern. The expression levels of *PelncRNA1* and its target genes were verified using qRT-PCR. The results demonstrated that the expression levels of *PelncRNA1* and its target genes increased during UV-B treatment. In *Arabidopsis* transgenic seedlings and moso bamboo protoplasts, *PelncRNA1* was discovered to influence the expression of its target genes when overexpressed. In addition, transgenic *Arabidopsis* showed higher tolerance to UV-B stress. These results suggest that *PelncRNA1* and its target genes are involved in the response of moso bamboo to UV-B stress. The novel findings would contribute to our understanding of how lncRNAs regulate the response to abiotic stresses in moso bamboo.

## Introduction

Moso bamboo (*Phyllostachys edulis*) is China’s most widely distributed and largest bamboo species (Ramakrishnan et al., 2020). As an essential urban green resource, moso bamboo has an exceptional capacity for carbon sequestration. Studies have demonstrated that the carbon sequestration capability of moso bamboo is significantly higher than that of other tree species, which is 2–4 times that of cedar (*Cunninghamia lanceolata*) (Yen, Ji & Lee, 2010; Yen & Lee, 2011). Therefore, the vast amount of bamboo forests can effectively fix carbon dioxide from the atmosphere and alleviate the greenhouse impact. However, the consequences of global climate change and frequent extreme weather have had various degrees of impact on plant growth, development, and photosynthesis. Specifically, the depletion of the ozone layer from the greenhouse effect has increased UV-B radiation at the surface. Zhang et al. (Feng et al., 2014; Zhang et al., 2011) found that excessive UV-B radiation causes chloroplast damage in plants, which substantially impairs the light-harvesting and electron-transfer capabilities of chloroplasts, resulting in a drop in net photosynthetic rate. Additionally, the strength of bamboo’s photosynthetic carbon sequestration ability is strongly related to its net photosynthetic rate (Zhizhuang et al., 2013). In summary, excessive UV-B radiation also affects bamboo’s ability to store carbon. Furthermore, higher UV-B radiation induces morphological changes in plants, such as decreased plant height and leaf area (Reddy et al., 2013), increased auxiliary branching (Li, He & Zu, 2010; Meijkamp, Doodeman & Rozema, 2001), and bronzing, chlorosis, and necrotic patches on leaves (Kakani et al., 2003). Therefore, it is essential to clarify the response mechanism of moso bamboo to UV-B stress and to enhance moso bamboo’s tolerance to UV-B stress for its growth, development, and photosynthesis.

Long non-coding RNA (lncRNA) is a type of transcript that is longer than 200 nucleotides (nt) but has no or low coding potential for translation into a protein (Kornienko et al., 2013; St Laurent, Wahlestedt & Kapranov, 2015). Initially, it was considered the “noise” generated by genomic transcription and did not have biological functions. However, many non-coding long-stranded RNAs have been discovered in humans, mice and other species, and their species, and their biological functions are presented one by one. There are four roles that lncRNAs play in molecular function: signals, decoys, guides, and scaffolds (Wang & Chang, 2011). Although only a tiny proportion of lncRNA functions have been identified, it has been demonstrated that lncRNA has transcriptional regulatory functions in organisms. Some researchers have found that lncRNA can promote staufen1 (STAU1) and mRNA binding and mediate their degradation (Gong & Maquat, 2011). Compared with mammals, plant lncRNA research is just beginning, but it has been found that lncRNA is involved in plant growth and development and environmental stress response. For example, low-temperature stress induces the expression of the antisense transcript *COOLAIR*, and increased *COOLAIR* will inhibit the expression of the *FLOWERING LOCUS C* (*FLC*) gene and thus participate in the vernalization of *Arabidopsis thaliana* (Groszmann et al., 2011; Swiezewski et al., 2009). Under the stress of hypoxia (Wu et al., 2012), light (Wang et al., 2014a), high temperature (Wunderlich, Gross-Hardt & Schoffl, 2014), and low phosphorus (Yuan et al., 2016) in *Arabidopsis*, high temperature (Xin et al., 2011) in wheat, and drought (Zhang et al., 2014) in maize, lncRNA is widely involved in abiotic stress. In moso bamboo, lncRNAs are also involved in the regulation of secondary cell wall (SCW) biosynthesis (Wang et al., 2021). However, no information is available about the involvement of lncRNAs in UV-B stress resistance in moso bamboo.

The noncoding RNA profile of moso bamboo was derived from the complete transcriptome database of four stressors, including low temperature, high temperature, ultraviolet light, and high salt (Ding et al., 2022). In this study, we selected one candidate lncRNA, which showed significant differential expression in moso bamboo seedlings after UV-B treatment. Then, we investigated the expression changes of the lncRNA and its target genes in bamboo protoplasts and *Arabidopsis* transformed with over-expression vectors of the lncRNA under UV-B treatment. The results show that the lncRNA and its target gene were involved in response to UV-B stress in moso bamboo.

## Material and Methods

### Plant material and UV-B treatment

The moso bamboo seeds were collected from a single mother bamboo. Seedlings with five mature leaves and consistent growth status were cultivated in a dark incubator (25 °C) for three days before treatment to eliminate the influence of UV in visible light (Biever, Brinkman & Gardner, 2014). For UV-B stress, UV-B treatments were applied for 2, 4, 6, and 8 h (Hunter et al., 2018; Makarevitch et al., 2015; Weber et al., 2020). The seedlings not subjected to the above stress treatments served as a control group (CK). All the samples were harvested directly into liquid nitrogen and stored at −80 °C until used for RNA extraction.

### Screening and identification of lncRNA

The annotation information of lncRNA was acquired from the whole transcriptome sequencing of moso bamboo (Ding et al., 2022). Based on this database, we screened lncRNA, and their target genes, which were differentially expressed under UV-B treatment. There are four software used to determine the coding of transcripts and identify typical lncRNAs, including CNCI (Coding On-Coding Index) (Sun et al., 2013), CPC2 (Coding Potential Calculator) (Kong et al., 2007), PLEK (predictor of long non-coding RNAs, and messenger RNAs based on an improved k-mer scheme), Pfam (PfamScan) (Finn et al., 2016).

### Target gene prediction and validation of lncRNA

There are two methods for predicting the target genes of lncRNAs. First, based on the position of genes near lncRNAs, we identified genes within 100 kb of the lncRNA as their cis-target genes with Perl scripts (Jia et al., 2010). The second method uses the online software LncTar (http://www.cuilab.cn/lnctar) to determine the normalized free energy according to how the bases of lncRNAs and mRNAs are paired (Li et al., 2015). Genes below the normalized free energy threshold were identified as trans-target genes of lncRNA.

Based on the transcriptome data, the differentially expressed target genes that corresponded to the relevant lncRNA transcription change trend were identified as the focus. QRT-PCR was used to confirm the relevance between *PelncRNA1* and its target gene expression trends, and weakly correlated target genes were eliminated.

### *PelncRNA1* cloning, vector construction

Two specific primers (Table S1) were designed to amplify *PelncRNA1*. Full-length *PelncRNA1* was cloned by PCR using KOD One™ PCR Master Mix (TOYOBO, KMM-101S, Shanghai, China). The positive PCR product was ligated into the transient over-expression vector pUBQ10 and the stable over-expression vector pER8, which were named pUBQ10-lncRNA and pER8-lncRNA respectively, and confirmed by Sanger sequencing. In the pUBQ10-lncRNA and pER8-lncRNA vectors, *PelncRNA1* was promoted by the 35S promoter.

### Protoplast isolation, polyethylene glycol (PEG) transfection, UV-B treatment

The protoplast isolation and PEG-mediated method were conducted as previously described (Hisamoto & Kobayashi, 2010; Yoo, Cho & Sheen, 2007), with some modifications. Briefly, 21-day-old moso bamboo leaf sheaths grown in soil were cut into small pieces using a razor blade and incubated for 4 h in an enzyme solution. After centrifugation, 2 − 3 × 10^4^ protoplasts were resuspended in a 200 µL MMG solution (4 mM MES-KOH [pH 5.7], 0.4 M mannitol, and 15 mM MgCl_2_). A total of 10 µg of pUBQ10-lncRNA were mixed well with 100 µL of protoplasts and a PEG solution (40% PEG4000, 0.8 M mannitol, and 1 M CaCl_2_). After 4 min of incubation, W5 solution (4 mM MES-KOH [pH 5.7], 0.5M mannitol, and 20 mM KCl) was added to the sample. The protoplasts were incubated and harvested.

Transfection was observed using a confocal laser scanning microscope (Zeiss, LSM510, Germany) after 12 to 16 h of incubation. Then, WT and transgenic protoplasts were exposed to UV-B radiation for 30 s. The protoplast viability would decrease if radiation exceeded 30 s (Fanguo & Guangmin, 2001; Gu, Lu & Yuan, 2004). For each treatment, three replicates were used for *PelncRNA1* and its target gene expression analyses.

### Genetic transformation and UV-B treated transgenic Arabidopsis

The constructs of pER8-lncRNA were transferred into *Agrobacterium tumefaciens* strain GV3101 and transformed into wide-type (WT) *Arabidopsis* (Col-0 ecotype) using the floral dip method (Zhang et al., 2006). The T0 transgenic plants were raised to adulthood. On half MS medium with 25 mg/L hygromycin, T1 seeds were harvested and germinated. Using *PelncRNA1*-specific primers, PCR was used to examine the segregation patterns in T1 progenies. T2 seeds from independent T1 lines showing 3:1 Mendelian segregation ratios were harvested separately and germinated on a hygromycin-containing rooting medium. T2 seedlings with absent segregation patterns were considered homozygous lines, and six were selected and used for subsequent UV-B stress experiments.

The 4-week-old transgenic and WT *Arabidopsis* were exposed to UV-B radiation for 1 and 2 h (Han & Han, 2015; You et al., 2011) to test their susceptibility to UV-B stress. Each treatment had three replicates, with natural daylight as the control group. The leaves of the samples were collected and immediately frozen in liquid nitrogen and stored at −80 °C until total RNA extraction was required.

### RNA extraction and qRT-PCR

Total RNAs were extracted with an RNA extraction kit (TIANGEN, DP441, Beijing, China). Reverse transcription was performed with the Hifair^®^ II 1st Strand cDNA Synthesis SuperMix for qPCR (YEASEN, 11123ES, Shanghai, China). Real-time quantitative PCR was performed on a standard protocol (CFX 96TM Touch Deep Well, BIO-RAD) using *NTB* and *ACTIN2* as a control. Primers for qPCR (Table S1) were designed with Primer3 (https://bioinfo.ut.ee/primer3-0.4.0/). The data was analyzed using the 2^−ΔΔCT^ method (Livak & Schmittgen, 2001).

### Phenotypic identification of transgenic *Arabidopsis* under UV stress

The malondialdehyde (MDA) and chlorophyll content of plant leaves were determined using the thiobarbituric acid colorimetric method and the SPAD-502plus chlorophyll meter (Minolta, Tokyo, Japan) after 0 h, 1 h, and 2 h of UV-B treatment, respectively. The single photon avalanche diode (SPAD) value is proportional to the relative content of chlorophyll (Ruiz-Espinoza et al., 2010). Three replicates were set up for each treatment, and the experimental results were calculated using IBM SPSS Statistics and Excel.

## Results

### Identification and analysis of *PelncRNA1* and its target gene

We screened the differentially expressed *PelncRNA1* and its target genes for differential expression in response to UV-B stress. It was identified as a long intergenic non-coding RNA (lincRNA) located in an intergenic region on chromosome 13 of the moso bamboo genome. It consists of one exon, and the full length of the transcript is 373 bp (Table S2). Then, CNCI, CPC2, PLEK, and Pfam were used to evaluate the authenticity of *PelncRNA1*. *PelncRNA1* cannot encode proteins, according to results from CNCI and Pfam software. *PelncRNA1* was also unlikely to encode proteins by CPC2 (Coding Probability: 0.0234985, Fickett Score: 0.40197) and PLEK (score −2.343590, RNA with a score less than 0 is considered non-coding RNA). Therefore, *PelncRNA1* is considered to be a typical non-coding RNA.

Next, the online software LncTar was used to calculate the normalized free energy of *PelncRNA1* and mRNA pairing sites. Genes below normalized free energy were identified as candidate target genes of *PelncRNA1* (Table S3). Furthermore, transcriptome data were used to filter the expression patterns of candidate genes that were consistent with or inverse to the expression modes of *PelncRNA1*. Finally, the differentially expressed candidate genes were chosen as the predicted target genes (Table S4), including PH02Gene33364 (Probable WRKY transcription factor 50), PH02Gene38550 (Wall-associated receptor kinase 3), PH02Gene43330 (Transcription factor BHLH148), PH02Gene19065 (CBL-interacting protein kinase 2), PH02Gene05460 (Purine permease 3), PH02Gene26812 (Berberine bridge enzyme-like 18), PH02Gene35897 (Chorismate synthase 2, chloroplastic), PH02Gene50461 (Hydroquinone glucosyltransferase). Details of the homologous genes of these target genes in *Arabidopsis* are listed in Table S4.

### Expression pattern of *PelncRNA1* and target genes in moso bamboo seedlings under UV-B stress

**Figure 1:**
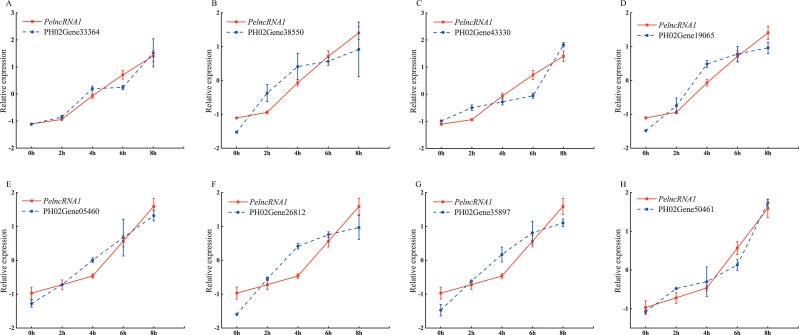
Expression pattern of PelncRNA1 and its predicted target genes under UV-B treatment. (A–H) Expression levels of *PelncRNA1* and its predicted target genes transcripts in moso bamboo, determined by qRT-PCR. The *y*-axis shows the relative expression levels analyzed by qRT-PCR. *X*-axis indicates UV-B irradiation time. Expression levels were normalized by the maximum value among samples and shown as mean ± standard deviation (*n* = 3) for *PelncRNA1* (red lines) and target genes (blue lines), respectively.

To study the expression pattern of *PelncRNA1* and its predicted target genes under UV-B treatment, we investigated the expression level of *PelncRNA1* and its predicted target genes under UV-B treatment at different times. The results showed that the *PelncRNA1* expression level increased during UV-B treatment (2 h, 4 h, 6 h, 8h). Moreover, the expression pattern of eight predicted target genes revealed a positive correlation with the expression level changes of the corresponding *PelncRNA1*. *PelncRNA1* was more strongly associated with its eight predicted target genes after UV-B treatment (Fig. 1).

### Over-expression of *PelncRNA1* in moso bamboo protoplasts

**Figure 2:**
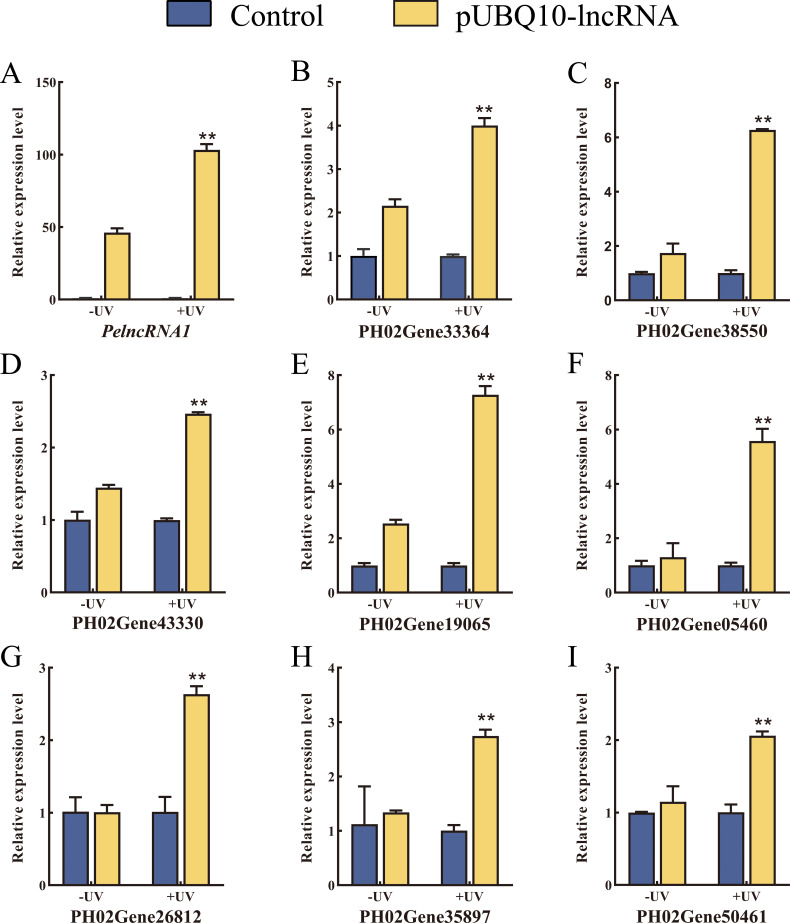
Over-expression of *PelncRNA1* in moso bamboo protoplasts. (A–I) The relative expression level of *PelncRNA1* and its target genes. The expression levels of *PelncRNA1* and its genes were measured by qRT-PCR normalized against the *NTB* gene. Bars and error lines indicate the mean ± standard error of three technical replicates; the independent sample *t*-test was used to determine the significant difference; * *p* < 0.05, ** *p* < 0.01.

To verify the authenticity of the expression pattern of *PelncRNA1* and its target genes under UV-B treatment, we constructed a pUBQ10-lncRNA vector (Fig. S2A) and transfected it into moso bamboo protoplasts by the PEG-mediated method (Fig. S2C). Compared with WT moso bamboo protoplasts, the relative expression level of *PelncRNA1* was significantly increased in protoplasts transfected with pUBQ10-lncRNA after UV-B treatment. Similarly, the relative expression level of target genes of *PelncRNA1* was also significantly increased (Fig. 2). The above results indicate that *PelncRNA1* and its target genes can respond to UV-B, and their expression patterns are positively correlated.

### Over-expression of *PelncRNA1* in *Arabidopsis*

We determined the non-homology of *PelncRNA1* by BLASTN analysis that no sequence similar to *PelncRNA1* was found in the other species, such as *Arabidopsis*, *Oryza sativa*, *Nicotiana tabacum*, *Glycine max*, and *Populus poplars*. This indicates that *PelncRNA1* is a moso bamboo-specific lncRNA. The full-length *PelncRNA1* transcript was cloned to construct a stable over-expression vector pER8-lncRNA (Fig. S2B). Ten independent transgenic lines were obtained, and six over-expression (OE-) lines were selected for further study. Using WT *Arabidopsis* as a negative control, PCR detection was performed using specific primers (Table S1). The results showed that six lines of transgenic *Arabidopsis* had successfully over-expressed *PelncRNA1* (Fig. S1).

**Figure 3:**
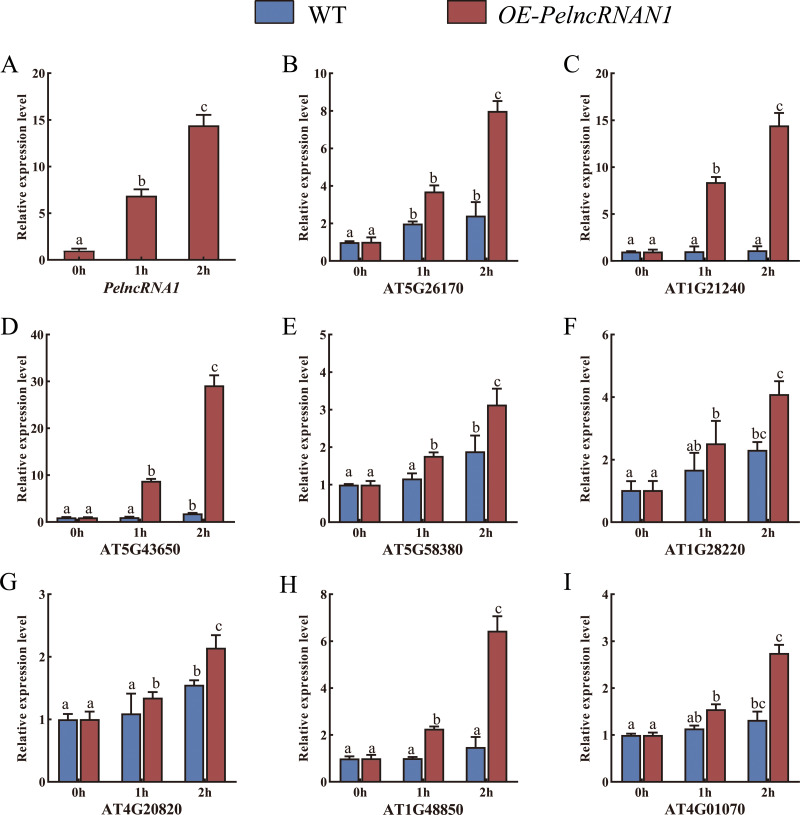
(A–I) Relative expression levels of target genes in WT and *OE-PelncRNA1 Arabidopsis* plants treated with UV-B. The gene expression levels were determined by RT-qPCR and normalized to the AtActin2 gene. The data are the mean values of three replicates ± standard deviation; treatment means followed by different lowercase letters vary significantly *p* = 0.05 in compliance with Fisher’s least significant differences (LSD) and Duncan’s multiple range test (DMRT) for multiple comparisons.

WT *Arabidopsis* and transgenic *Arabidopsis* T2 plants (*OE-PelncRNA1*) were treated with UV-B for 1 h and 2 h. In *OE-PelncRNA1* plants, the expression level of *PelncRNA1* was significantly up-regulated after UV-B stress treatment (Fig. 3A). Under untreated conditions, the expression levels of these target genes in *OE-PelncRNA1* plants were similar to those in WT *Arabidopsis*. However, the expression of these genes increased in both WT and *OE-PelncRNA1* plants after UV-B stress treatment (Figs. 3B–3I). The expression levels of target genes in *OE-PelncRNA1* plants were significantly higher than that of WT after UV-B treatment, especially AT5G43650 (Fig. 3D). These data indicated that *PelncRNA1* could regulate plant tolerance to UV-B stress by regulating the expression of homologous target genes.

**Figure 4:**
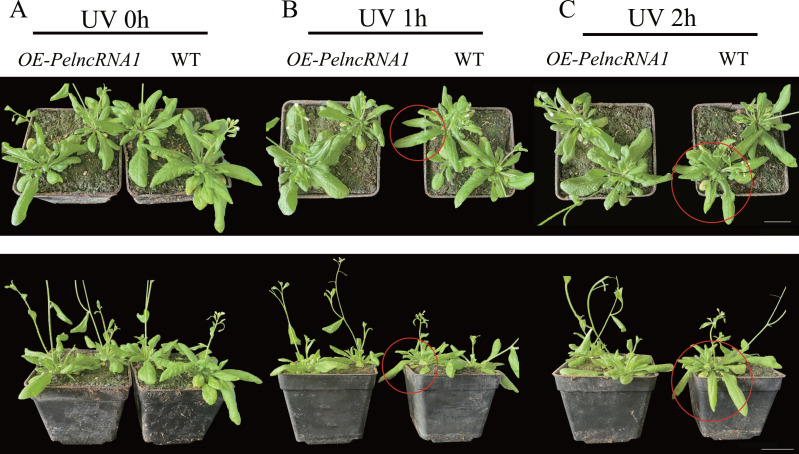
The phenotype of overexpressed *PelncRNA1* plants under UV-B stress treatment. (A) natural light group; (B) 1 h UV-B treatment; (C) 2 h UV-B treatment. Using WT plants as a control, OE-PelncRNA1 plants showed tolerance to UV-B stress. The circularly marked parts are the leaves with more obvious wilting.

Additionally, phenotypic observation found no abnormal phenotype of *OE-PelncRNA1* under natural light compared to WT (Fig. 4A). After one hour of UV-B treatment, *OE-PelncRNA1* leaves did not wilt or lose water, but WT leaves started to wilt lightly (Fig. 4B). After 2 h of treatment, the leaves of *OE-PelncRNA1* were only slightly wilted, whereas the WT leaves were severely wilted and significantly damaged by UV-B radiation (Fig. 4C). This result demonstrates that *PelncRNA1* increases the resistance of transgenic *Arabidopsis* to UV stress.

**Figure 5:**
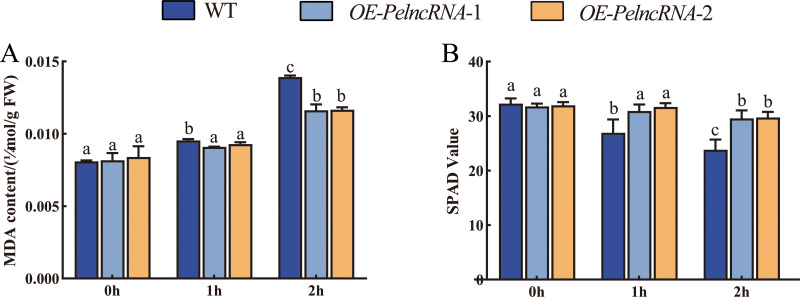
Plant MDA content and chlorophyll content. (A) MDA content. *Y*-axis indicates MDA content (µmol/g FW). (B) Relative chlorophyll content. *Y*-axis indicates the SPAD (single-photon avalanche diode) value that was proportional to the relative content of chlorophyll. The *X*-axis indicates UV-B irradiation time. Duncan’s multiple range test (DMRT) and least significant difference (LSD) test were used to identify differences between means. The significance level was *p* < 0.05; different letters indicated significant differences between treatments.

We also examined the malondialdehyde (MDA) and chlorophyll content of transgenic *Arabidopsis* with *OE-PelncRNA1* under UV-B stress. Under natural light conditions, the contents of MDA and chlorophyll in *OE-PelncRNA1* and WT lines were similar. However, after 1 h UV-B treatment, the MDA content of *OE- PelcRNA1* was not significantly different but significantly increased in WT. MDA was increased in both *OE-PelncRNA1* and WT plants after 2 h treatment, but the elevation in WT plants was much larger than in *OE-PelncRNA1* plants (Fig. 5A). The content of MDA concentration can represent the degree of cell membrane lipid peroxidation and is a crucial indication for detecting plant stress (Tsikas, 2017). The result implies that *OE-PelncRNA1* plants had lower membrane lipid peroxidation under UV-B stress than WT plants.

After UV-B treatment, both *OE-PelncRNA1* and WT plants’ SPAD values (single photon avalanche diode) decreased, and the SPAD value of WT plants decreased much more than the *OE-PelncRNA1* plants (Fig. 5B). Since the SPAD value was proportional to chlorophyll content, this experiment demonstrated that the UV-B treatment on chlorophyll in *OE-PelncRNA1* plants was less adverse than in WT plants. The physiological and phenotypic observations confirmed that *OE-PelncRNA1* plants were more tolerant to UV-B stress than control plants.

## Discussion

Long non-coding RNA (lncRNA) is an RNA incapable of encoding proteins. With the rapid development of biotechnology, there is increasing evidence that lncRNAs are potential regulatory molecules. LncRNAs can act as a scaffold molecule (Ariel et al., 2014), an inducer molecule (Bardou et al., 2014), a guide molecule (Crespi et al., 1994), and a signal molecule (Swiezewski et al., 2009). It influences gene expression at the transcriptional, post-transcriptional, and epigenetic levels (Mercer, Dinger & Mattick, 2009), thereby regulating the life activities of various plants to resist drought (Tan et al., 2020), salinity (Qin et al., 2017), low phosphorus (Wang et al., 2017), low temperature (Moison et al., 2021), and other abiotic stresses. However, few studies have focused on the response mechanism of lncRNA involved in UV-B stress.

In this study, a novel intergenic lncRNA, *PelncRNA1*, was identified to be associated with UV-B stress responses in moso bamboo. The analysis of coding potential showed that *PelncRNA1* was a typical lncRNA with no protein-encoding potential; this is the same as most lncRNAs found (Rinn & Chang, 2012). Like many other lncRNAs, the basic transcript level of *PelncRNA1* was low, but its expression was increased after UV-B treatment. In addition, we used the online software PlantCARE (http://bioinformatics.psb.ugent.be/webtools/plantcare/html/) to analyze the sequence of the promoter region of 2 kb in length upstream of the transcription start site. We found that the *PelncRNA1* promoter contains cis-acting elements in response to gibberellin, abscisic acid, stress, and light, suggesting UV-related stress may induce *PelncRNA1* (Table S7, Fig. S3), further indicating that *PelncRNA1* may be induced by UV-B stress. Up to date, only a few lncRNAs are partially evolutionarily conserved in animals and plants, and most lncRNAs are not homologous among species (Liu, Wang & Chua, 2015; Marques & Ponting, 2009). Similarly, homologous *PelncRNA1* was not identified in other species, such as rice, *Arabidopsis thaliana*, etc.

Many studies have shown that lncRNA can activate or inhibit gene transcription by *cis* or *trans* regulation (Kopp & Mendell, 2018). For example, in *Arabidopsis*, lncRNA *HIDDEN TREASURE 1* (*HID1*) is directly involved in the transcription of the *PHYTOCHROME-INTERACTING FACTOR 3* (*PIF3*) gene, a key inhibitor of photomorphogenesis, primarily by trans-acting (Wang et al., 2014b). In addition, lncRNA33732 in tomatoes acts as a positive regulator and enhances tomatoes’ resistance to *Phytophthora infestans* by induction of the expression of respiratory burst oxidase (RBOH) and increase in the accumulation of H_2_O_2_ (Cui et al., 2019). In the study, we found that the target genes of *PelncRNA1* are located far from it, and the expression pattern of *PelncRNA1* and its target gene is positively correlated. We hypothesized that *PelncRNA1* might regulate the expression of its target genes through a transaction.

Abiotic stresses, such as salt and drought, significantly impact plant growth and development. Plants usually need to pa growth and yield costs to cope with abiotic stresses. Many lncRNAs have been identified in plants in response to abiotic stress. For example, *CBF1* (C-repeat/dehydration-responsive element binding factors) is a gene that responds to low-temperature stress. LncRNA *SVALKA* is located in the neighboring region of *CBF1*, and *SVALKA* can repress the transcription of *CBF1* and thus regulate cold plant tolerance (Kindgren et al., 2019). *Arabidopsis* lncRNA *AUXIN-REGU-LATED PROMOTER LOOP* (*APOLO*) can regulate root hair elongation in response to low temperature by regulating H3K27me3 deposition in the *ROOT HAIR DEFECTIVE 6* (*RHD6*) promoter region and WRKY42 interactions to regulate the opening of the promoter loop (Moison et al., 2021).

WRKY and BHLH are a family of transcription factors widely present in plants. Their biological functions are divers (Qian et al., 2021; Rushton et al., 2010; Wang et al., 2019), including involvement in plant growth and development, senescence, and response to adversity stress. Studies have shown that transcription factors WRKY and BHLH are involved in the process of photomorphogenesis. For example, the transcription factor WRKY DNA-binding protein 36 (WRKY36) was found to be a repressing regulator of ELONGATED HYPOCOTYL5 (HY5) transcription and UV-B photomorphogenesis. In response to UV-B radiation, the plant-sensitive UV-B-specific photoreceptor UV RESISTANCE LOCUS8 (UVR8) homodimers monomerize instantaneously to active monomers, enter the nucleus, and inhibit the DNA-binding capacity of the WRKY36 transcription factor, thereby promoting HY5 transcription, inhibiting hypocotyl elongation and promoting photomorphogenesis (Fernández, Lamattina & Cassia, 2020). In strawberries, FvHY5 promotes anthocyanin synthesis by forming a heterodimer with FvbHLH9 to activate the expression of FvDFR (gene15176) (Li et al., 2020). However, information on the expression and function of the WRKY and BHLH transcription factor families in response to UV-B stress is very limited. In this study, the expression of WRKY50 and BHLH92 transcription factors increased under UV-B treatment. We speculated that WRKY50 and BHLH92 might respond to UV-B stress by participating in the photomorphogenesis pathway.

In addition, in this study, the expression levels of WAK (Cell wall-associated receptor kinase), CIPK (Calcineurin B like protein interacting protein kinase), PUP (purine permease), and CS (chorismate synthase) were significantly increased under the induction of UV-B. Previous studies have shown that WAK is connected to the cell wall and is an important protein connecting plants’ cell walls and cytoplasm (Wang et al., 2012). CIPK is a plant-specific serine/threonine protein kinase, which belongs to the third class of SnRK3 kinases (SNF-1 related protein kinase 3, SnRK3) (Guo et al., 2001; Ohta et al., 2003). PUP is a protein with the function of transporting cytokinin (Qi & Xiong, 2013). All of the above can participate in plant defense and stress. For example, the expression of *QsCIPK3* in rice crops is up-regulated after low-temperature induction, and overexpression of *QsCIPK2* can enhance the drought tolerance of rice (Xiang, Huang & Xiong, 2007). Wheat *TaCIPK7*, *TaCIPK15*, *TaCIPK24*, and *TaCIPK32* genes are involved in the induction of plant low-temperature stress response (Sun et al., 2015). Rice *OsPUP7* plays a role in cytokinin transport, affecting rice growth, development, and stress response (Qi & Xiong, 2013). The same CS is involved in the shikimic acid pathway, localized in chloroplasts, and in plant growth and development. For example, gene silencing of PhCS in petunia decreased CS activity, further leading to growth retardation, abnormal flower, leaf development, and decreased folic acid (including chlorophyll, carotenoid and anthocyanin) levels (Zhong et al., 2020). In summary, we speculate that *PelncRNA1* may improve the tolerance of moso bamboo to UV-B by inducing the expression of these genes related to plant stress resistance. However, the specific regulatory mechanism must be clarified and further studied.

## Conclusions

The rapidly increasing number of plants lncRNAs and their multifaceted regulatory roles governing various biological processes is becoming a hotspot in biological research (Budak, Kaya & Cagirici, 2020; Nejat & Mantri, 2018). However, there is still much to be studied about the function and mechanism of lncRNA, especially in moso bamboo. In this study, we identified a novel intergenic lncRNA, *PelncRNA1*, related to UV-B stress based on the whole transcriptome database of moso bamboo. It was found that both *PelncRNA1* and its target genes could respond to UV-B, and the expression pattern was positively correlated. In addition, we found that plants overexpressing *PelncRNA1* showed tolerance to UV-B stress. Therefore, *PelncRNA1* could enhance plant tolerance to UV-B stress by regulating the expression of transcription factors related to the UV-B signaling pathway and genes associated with plant abiotic stress. This study provides new knowledge of the involvement of lncRNA in the abiotic stress response of moso bamboo. It also provides a strategy for cultivating new bamboo species with strong stress resistance.

##  Supplemental Information

10.7717/peerj.15243/supp-1 Figure S1 *OE-PelncRNA1* transgenic *Arabidopsis* identification M: DL5000 DNA Marker; 1-10: OE-*PelncRNA1* transgenic *Arabidopsis*; 11-14: Wild-type *Arabidopsis*

10.7717/peerj.15243/supp-2 Figure S2 Moso bamboo protoplast isolation and transient gene expression A. Plasmid map of transient over-expression vector pUBQ10-lncRNA; B. Plasmid map of stable over-expression vector pER8-lncRNA; C. Expression of EGFP (Enhanced Green Fluorescent Protein) in moso bamboo protoplasts. Experiments were repeated 2–3 times. Scale bar = 20 µm.

10.7717/peerj.15243/supp-3 Figure S3 The promoter analysis of *PelncRNA1* The 2-Kb DNA fragment upstream of *PelncRNA1* was analyzed using PlantCARE.

10.7717/peerj.15243/supp-4 Table S1 The primers used for qRT-PCR

10.7717/peerj.15243/supp-5 Table S2 Full-length sequence of *PelncRNA1*

10.7717/peerj.15243/supp-6 Table S3 The normalized free energy of PelncRNA1 and mRNA pairing sites

10.7717/peerj.15243/supp-7 Table S4 Gene information

10.7717/peerj.15243/supp-8 Table S5 SPAD value and MDA content

10.7717/peerj.15243/supp-9 Table S6 Quantification Cq Results

10.7717/peerj.15243/supp-10 Table S7 Promoter prediction by PlantCARE

## Additional Information and Declarations

### Data Availability

The following information was supplied regarding data availability: The raw data is available in the Supplemental Files.
