# Supplementary figures and images for: A long non-coding RNA PelncRNA1 is involved in Phyllostachys edulis response to UV-B stress
Source: PeerJ. 2023 May 9;11:e15243. doi: 10.7717/peerj.15243 (PMC10178214; doi:10.7717/peerj.15243)

A

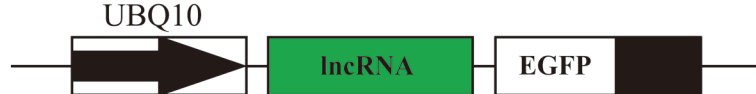

B

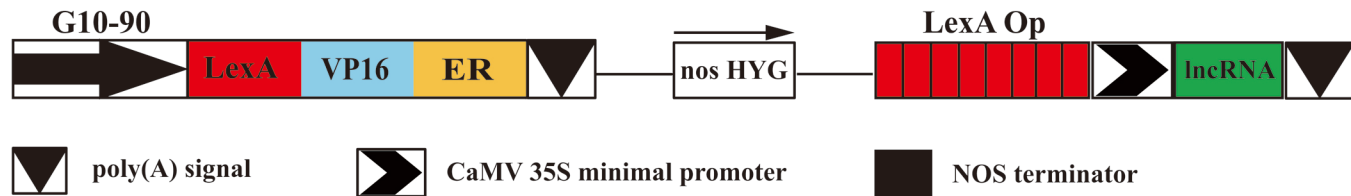

C

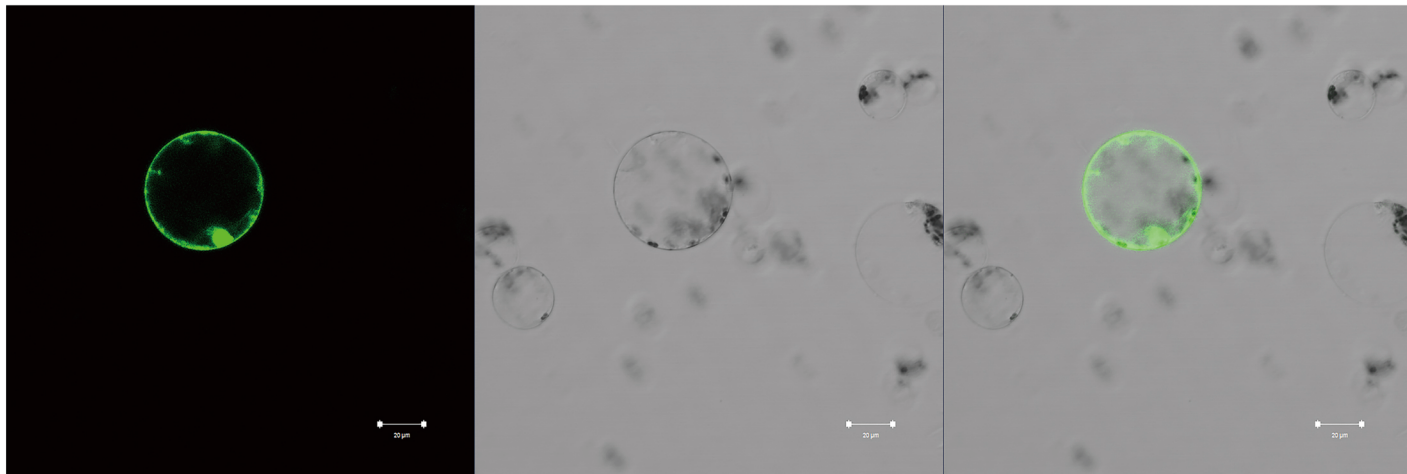

Supplement: Figure S2 — A. Plasmid map of transient over-expression vector pUBQ10-lncRNA; B. Plasmid map of stable over-expression vector pER8-lncRNA; C. Expression of EGFP (Enhanced Green Fluorescent Protein) in moso bamboo protoplasts. Experiments were repeated 2–3 times. Scale bar = 20 µm. [file peerj-11-15243-s002.pdf]

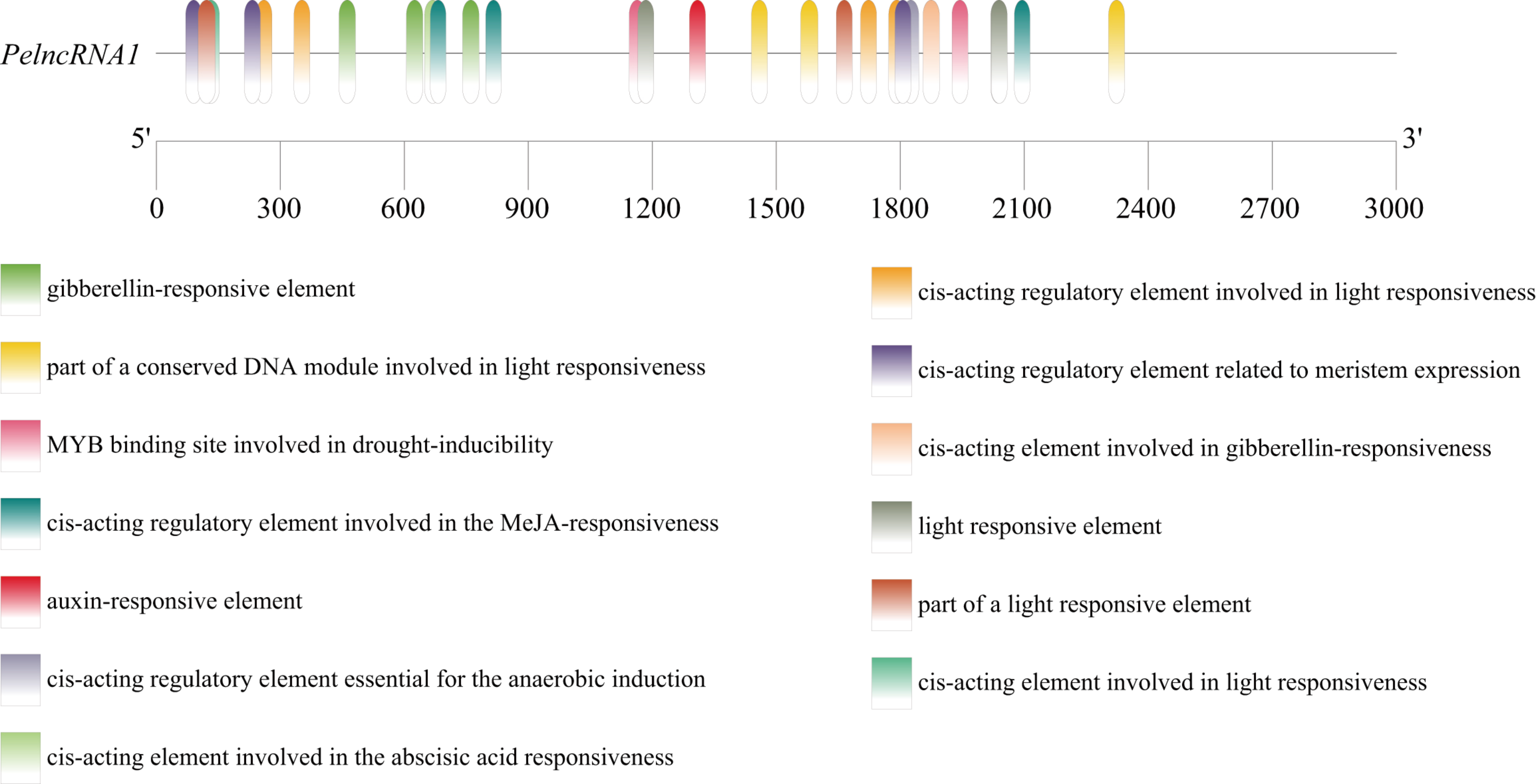

Supplement: Figure S3 — The 2-Kb DNA fragment upstream of PelncRNA1 was analyzed using PlantCARE. [file peerj-11-15243-s003.pdf]
