# Supplementary material for: A long non-coding RNA PelncRNA1 is involved in Phyllostachys edulis response to UV-B stress
Source: PeerJ. 2023 May 9;11:e15243. doi: 10.7717/peerj.15243 (PMC10178214; doi:10.7717/peerj.15243)
Supplement: Table S2 [file peerj-11-15243-s005.docx]

**Table S2 Full-length sequence of *PelncRNA1***

> *PelncRNA1*_geneID=XLOC_234827_moso_draft_hic_scaffold_13_43954912 43955284

TTCACAGCAACACTAGTGGTAACATAGCAACAACACAAGCTTTATTAATAACCAAAGGAGTTGTTTACAAGAGAGCTTACAAGCCAGTAGACAAAACATAGAAATATAAACACATGAGCCACCCGATACACCACAGGCAACCGGCTGGAGGGCGCAATCCTAGTCGATCTGGACCATTTGGTCTACAGAACTGGTCACCCAAAGCTTCCATAACCTGCTAAAGACAGCATCAAGCAAGAGTGAGCACAACAACCGTGCTCGCAAGTTACACCCGTCAGATAACAAAATGACATGCATAAATGGATATCACAATGGAAGGCTTATTATGGTTAATTTGCATAAAGCCATGTTTATTTCCAATTACCAGTATTAA
